# Prevalence of Pre Diabetes and Type 2 Diabetes Mellitus among cement industry workers

**DOI:** 10.12669/pjms.36.2.1266

**Published:** 2020

**Authors:** Sultan Ayoub Meo, Yasser Abdullah Bin Muneif, Nasser Abdullah BenOmran, Mohammad Abdullah AlSadhan, Raed Fuad Hashem, Abdullah Saud Alobaisi

**Affiliations:** 1 Dr. Sultan Ayoub Meo, MBBS, PhD. Department of Physiology, College of Medicine, King Saud University, Riyadh, Saudi Arabia; 2 Dr. Yasser Abdullah Bin Muneif, MBBS. Department of Physiology, College of Medicine, King Saud University, Riyadh, Saudi Arabia; 3 Dr. Nasser Abdullah BenOmran, MBBS. Department of Physiology, College of Medicine, King Saud University, Riyadh, Saudi Arabia; 4 Dr. Mohammad Abdullah AlSadhan, MBBS. Department of Physiology, College of Medicine, King Saud University, Riyadh, Saudi Arabia; 5 Dr. Raed Fuad Hashem, MBBS, Department of Physiology, College of Medicine, King Saud University, Riyadh, Saudi Arabia; 6 Dr. Abdullah Saud Alobaisi, MBBS. Department of Physiology, College of Medicine, King Saud University, Riyadh, Saudi Arabia

**Keywords:** Cement dust, Diabetes mellitus, Occupational settings, Prevalence

## Abstract

**Objectives:**

Occupational and environmental pollution have become an imperative jeopardy for developing devastating metabolic diseases. Limited animal model studies have examined the impact of exposure to cement dust on metabolic conditions. This study aimed to assess the prevalence of pre-diabetes and Type-2 diabetic mellitus (T2DM) among non-smoking cement mill workers.

**Methods:**

This epidemiological cross sectional study was conducted in the “Department of Physiology, College of Medicine, King Saud University, Riyadh, Saudi Arabia” during the period Oct 2016 to June 2017. Initially 310 cement mill workers were interviewed; after the interview and clinical history taking, 186 non-smoking cement mill employees were finally recruited. The cement mill employees were exposed to cement dust-related pollution in a cement industry for eight hours a day for six days a week. The mean age was 36.56 ± 0.78 years, mean BMI was 25.70 ± 0.29 m/kg2, and mean period of employment in the cement industry was 82.77 ± 6.95 months. HbA1c was measured using the Dimension Xpand Plus Integrated Chemistry System (USA).

**Results:**

The cement mill employees were divided into three groups: non-diabetics group, with glycated hemoglobin (HbA1c) <5.7%; pre-diabetics group, with HbA1c 5.7-6.4%; and diabetics group, with HbA1c >6.4%. Among the cement mill personnel, 79 (42.47%) were non-diabetics, 28 (15.05%) were pre-diabetics, and 79 (42.47%) were diabetics. The prevalence of pre-diabetes and T2DM among cement mill employees was considerably associated with the period of employment in the cement industry (p=0.032).

**Conclusions:**

Exposure to cement dust was associated with an increased prevalence of pre- diabetes and T2DM among cement industry employees.

## INTRODUCTION

Diabetes mellitus (DM) is a growing global community concern with multiple deplorable complications. Despite notable developments in health sciences, DM remains an incorrigible chronic illness.^1^ It involves and impairs multiple essential physiological functions^2^, body systems^3^ and accompanies different devastating complications.^4^ It is swiftly growing in developing and developed nations and has penetrated both rural and urban regions. About 425 million people worldwide are living with DM, and 212 million individuals with diabetes remain undiagnosed. About 279 million people are living in urban areas, while 146 million live in rural areas. Meanwhile, 327 million people with diabetes are at the peak of their waged age.^5^

Environmental pollution is a global concern as it is associated with a wide range of adverse health outcomes.^6^ Globally, millions of individuals are working in industrial sectors, including cement factories, and are exposed to dust at various stages of the production processes. Cement dust mainly consists of “calcium oxide, silicon oxide, aluminum trioxide, ferric oxide, magnesium oxide, sand, and other impurities”.^7^,^8^ Exposure to cement dust remains an emerging problem and contributes to the development of respiratory9 and coronary artery diseases.^10^-^12^ Industrial workers who are frequently exposed to dust develop insulin resistance, glucose metabolism dysfunction, and Type-2 diabetic mellitus (T2DM).^13^,^14^ . Literature is acutely lacking to find out the prevalence of T2DM among cement industry workers. The existing studies primarily utilized animal models; therefore, this study, which is the first of its kind, aimed to assess the prevalence of pre-diabetes and T2DM among non-smoking male cement industry workers.

## METHODS

### Selection of Participants

This epidemiological cross sectional study was conducted in “Department of Physiology, College of Medicine, King Saud University, Riyadh, during the period Oct 2016 to June 2017. Cement industry workers who voluntarily participated and had same age, gender, ethnicity, and socioeconomic background were selected. It was ensured that the cement industry workers had no previous history of working in plastic, steel, wood, welding, oil, and cotton factories. Initially, 310 cement mill employees of same exposure and duration levels were interviewed. These workers did not use personal protective measure; after history taking and examination, 186 (60%) non-smoking cement mill employees were recruited and 124 (40%) were excluded from the study. A large number of participants were excluded as they had a history of cigarette smoking, different socioeconomic condition, previous history of working in manufacturing factories other than cement, and had health issues.^15^,^16^ The cement industry employees were exposed to cement dust for eight hours a day for six days a week. The mean age was 36.56 ± 0.78 years, mean BMI was 25.70 ± 0.29 m/kg^2^, and mean employment duration in cement industry was 82.77 ± 6.95 months. A statistical power analysis was conducted to determine the sample size; within the anticipated prevalence of T2DM, the targeted population sample size was about 4,000 with 5% margin of error and 95% confidence level. The required sample size was 255, but only 186 cement factory workers were recruited in this study.

### Exclusion Criteria

Cement mill employees with a known history of “anemia, blood transfusion, obesity, and asthma; personal or family history of DM; and malignancy” were excluded.^15^ Cement mill employees who smoked cigarette or shisha^17^ and previously worked in other manufacturing factories that produce dust or fumes were excluded, as smoking^18^ and dust increase the peril for DM.^15^,^16^

### Blood Sample Collection

Cement mill workers were assigned a number; a 2-ml of blood was obtained by a para-medical staff member through a vein puncture procedure and was placed in a container with ethylene diamine tetra-acetic acid. The identification number of employees was placed on the container, and the container was transported to the laboratory for the analysis of glycated hemoglobin (HbA1c).^14^

### Measurements of Glycated Hemoglobin

The HbA1c was used to measure glycemic control within a period of 3-4 months. HbA1c played an essential role in the diagnosis of DM19. HbA1c was measured using the “Dimension Xpand Plus Integrated Chemistry System (USA)”.^14^ The “American Diabetes Association (ADA)” approach was applied; based on HbA1c levels, cement industry workers were divided into three groups. Cement industry workers with HbA1c <5.7% were classified as non-diabetics, those with HbA1c 5.7%–6.4% as pre-diabetics, and those with HbA1c >6.4% as diabetics.^19^

### Ethics Statement

The study was approved by the Institutional Review Board College of Medicine Reacher Centre, King Saud University, Riyadh, Saudi Arabia (E 18-3654), written consent was obtained from the cement industry worker.

### Statistical Analysis

The program SPSS Version 22 Microsoft Windows was used. Continuous variables were presented as means ± standard deviations, while descriptive data were presented as numbers and percentages. The relationship between sociodemographic and period of employment in the cement factory was calculated using χ^2^ tests of independence. The significance level was presumed at p<0.05.

## RESULTS

The sociodemographic physiognomies of the workers are presented in Table-I. Based on the 2018 ADA guidelines, the cement industry workers were divided into three groups: nondiabetics (HbA1c <5.7%), pre-diabetics (HbA1c 5.7%–6.4%), and diabetics (HbA1c >6.4%. About 79 (42.47%) workers were nondiabetics, 28 (15.05%) were pre-diabetics, and 79 (42.47%) were diabetics (Table-II).

**Table-I:** Sociodemographic characteristics of cement industry workers (n=186).

Variables	Mean (SDE)
Age (years)	36.56 ± 0.78
BMI (m/kg)2	25.70 ± 0.29
Exposure: months	82.77 ± 6.95
HbA1c %	6.31 ± 0.10

Values are expressed in mean ± SDE, BMI: Body Mass Index.

**Table-II:** Prevalence of pre-diabetes and T2DM in cement industry workers (n=186).

Parameters	Numbers and Percentage
Normal: HbA1c < 5.7%	79 (42.47%)
Pre-diabetic: HbA1c 5.7-6.4 %	28 (15.05%)
Diabetic: HbA1c > 6.4%	79 (42.47%)

***Note:*** HbA1c values are presented based on American Diabetic Association Guidelines 2018.

T2DM = type 2 diabetes mellitus; HbA1c = glycated hemoglobin.

The prevalence of pre-diabetes and T2DM among cement mill workers was associated with employment duration in cement industry (Table-III). The mean duration of employment for nondiabetics was 65.49±8.41 months, that for pre-diabetics was 72.42±17.97 months, and that for diabetics was 103.72±12.24 months. The period of employment in cement industry was significantly associated with T2DM (p=0.032) (Table-II). The mean BMI of nondiabetics was 25.60 ± 0.48 m/kg2, that of pre-diabetics was 26.60 ± 0.75 m/kg2, and that of diabetics was 25.48 ± 0.40 m/kg2. There was no significant association between the BMI and T2DM (p=0.421) (Table-III).

**Table-III:** Prevalence of pre-diabetes and T2DM in cement industry workers (n=186).

Variables	Non-diabetics (n=79)	Pre-diabetics (n=28)	Diabetics (n=79)	F-value	P- value
Parameters	HbA1c < 5.7%	HbA1c 5.7-6.4 %	HbA1c > 6.4%		
Age (years)	33.64 ± 0.96	37.25 ± 1.82	39.24 ± 1.39	5.68	0.004
BMI (m/kg)2	25.60 ± 0.48	26.60 ± 0.75	25.48 ± 0.40	0.87	0.421
Exposure: months	65.49 ± 8.41	72.42± 17.97	103.72 ± 12.24	3.49	0.032

***Note:*** Values are expressed in mean ± SD. HbA1c values are presented based on American Diabetic Association Guidelines 2018. BMI = Body Mass Index; SD = standard deviation; HbA1c = glycated hemoglobin.

The age of non-diabetic’s workers was 33.64 ± 0.96 years; pre-diabetics, 37.25 ± 1.82 years; and diabetics, 39.24 ± 1.39 years. There was a significant association between age and T2DM (p=0.004) (Table-III). The association between the occurrence of pre-diabetes and T2DM with age and duration showed that employees who worked in a cement industry for a longer duration had higher age than those who worked in the same industry for a shorter duration.

## DISCUSSION

DM is a leading health concern and its prevalence is increasing globally. DM has been previously documented to be due to genetics, sedentary lifestyles and unhealthy food habits. This novel study identified that the prevalence of pre-diabetes and T2DM significantly increased in cement industry workers.

Wang et al. (2014)20 identified that prolonged exposure to air pollution increases the risk of T2DM. Balti et al. (2014)21 investigated the association between pollutants and the occurrence of DM. The study showed that dust pollutants have a significant effect on the occurrence of DM. Park and Wang (2014)22 performed a systematic appraisal on air pollution and T2DM and showed that air pollution is a risk factor for TD2M. In another study, Eze et al. (2015)23 reported that persistent exposure to air pollution causes DM. Similarly, Weinmayr et al. (2015)24 reported that long-term exposure to road traffic pollution increases T2DM risk in general population.

Meo et al. (2018)15 piloted a study in occupational settings and reported that the prevalence of pre-diabetes and T2DM was significantly augmented among plastic industry employees. In another study, Meo et al. (2015)2 identified that air pollution is the main reason for the occurrence of insulin resistance and T2DM. This study identified that prevalence of pre-diabetes and T2DM among cement mill workers was significantly increased. The possible reason for this is that cement industry-associated dust pollution causes insulin resistance and ultimately lead to the development of DM.

Kelsall et al. (2018)25 determined TD2M and risk in the occupational and industry working population. In comparison to office workers and technical service providers, increased prevalence of DM was reported in numerous occupational groups who were employed in dust-generating factories. Moreover, blue-collar industry workers had a higher risk of DM. The occupations in which workers are exposed to high pollution are associated with a greater risk of DM. The effect of occupational pollutants and the mechanism underlying the onset of T2DM are very complex. The potential underlying mechanisms behind occupational exposure and T2DM are inflammation and insulin resistance. The occupational-associated pollutants contribute to oxidative stress, low-grade inflammation, insulin resistance, glucose metabolism impairment and TD2M.

### Strengths of the study

This is the first human model study to examine the prevalence of pre-diabetes and T2DM among employees of the cement industry. The study exclusion criteria were highly consistent, and the study excluded smokers.

### Limitations of the study

Although, it was attempted to recruit an adequate number of employees from a cement industry, most of the workers were cigarette smokers, obese, and differed in socioeconomic conditions; hence, a limited number of cement industry workers were recruited and evaluated to determine the association between exposure to cement dust and prevalence of pre-diabetes and T2DM.

## CONCLUSIONS

Occupational exposure to cement dust is associated with an increased prevalence of pre-diabetes and T2DM. The occupational health administrators must provide preventive measures to minimize the pollution from the cement factories in order to protect the workers and provide healthier industrial allied work-related conditions.

### Authors’ Contributions:

**SAM:** Study design, supervised the project, data analysis and manuscript writing.

**YAM, NAO, MAA, RFH, ASA:** Literature review, subject selections and data collection,

All authors have approved the final version of the manuscript.
